# A new species of *Leiochrides* from the Korean subtidal waters with notes on the taxonomic status of the genus *Pseudomastus* (Annelida, Capitellidae)

**DOI:** 10.3897/zookeys.685.12700

**Published:** 2017-07-17

**Authors:** Man-Ki Jeong, Jin Hee Wi, Hae-Lip Suh

**Affiliations:** 1 Department of Oceanography, Chonnam National University, Yongbong-ro, Buk-gu, Gwangju 61186, Korea; 2 School of Environmental Science and Engineering, Gwangju Institute of Science and Technology, Cheomdangwagi-ro, Buk-gu, Gwangju 61005, Korea

**Keywords:** Korea, *Leiochrides yokjidoensis* sp. n., morphology, Polychaeta, Scolecida, Sedentaria

## Abstract

*Leiochrides yokjidoensis* **sp. n.**, collected from the sublittoral muddy bottom in southern Korea, is described as a new species. The taxonomic status of the monospecific genus *Pseudomastus* has been a subject of controversy for many years, as its characteristics overlap those given in recent generic definitions of *Leiochrides*. The results of a comprehensive review and comparison regarding the two genera, based on previous records showed minor differences. In this study, a detailed description of *L. yokjidoensis* **sp. n.** is given and a comparison with closely related species is tabulated and discussed.The taxonomic status of *Pseudomastus* is discussed and the genus placed in synonymy with *Leiochrides*.

## Introduction

Polychaetes are an important component of the macrobenthic community and they play a crucial role in the functioning of benthic communities in the recycling and reworking of the benthic sediments, bioturbation, and in the burial of organic matter (Hutchings 1998). The Capitellidae Grube, 1862 are found in many types of sediments from the intertidal region to the deep sea and is a frequently dominant component of the benthic infaunal communities, especially in organically enriched sediments (Blake 2000). Owing to their accessibility and their importance in sedimentary environments, capitellids have been the subject of numerous ecological studies (Blake 2000). Despite their ecological importance, the taxonomy of Capitellidae has been largely ignored (Dean 2001). Since the first designation by Grube (1862), the family Capitellidae is currently composed of 48 genera, including 24 monospecific genera (Read 2017). The genera in this family have been defined by the number of segments with capillary chaetae or hooded hooks or mixtures of both (Blake 2000, Fauchald 1977). However, several previous records of Capitellidae include incorrect taxonomic information due to the ontogenetic variations in the chaetal arrangement and an indistinct demarcation of the peristomium or the transitional segment (Blake 2000, Green 2002, García-Garza and León-González 2011). Moreover, the high percentage of monospecific genera and the use of the insufficient characters in identification keys (i.e. based mostly on segmental distribution and chaetal composition) have steadily raised questions about taxonomic concepts of many Capitellidae (Ewing 1991, Blake 2000).

The genus *Leiochrides* was established by Augener (1914) with the description of *L. australis* Augener, 1918 from southwest Australia. The additional seven described species in the genus are *L. africanus* Augener, 1918 from western Africa, *L. pallidior* (Chamberlin, 1918) and *L. biceps* Hartman, 1954 from California, *L. hemipodus* from Southern California, *L. norvegicus* Fauchald, 1972 from western Norway, and *L. branchiatus* Hartman, 1976 and *L. andamanus* Green, 2002 from the Indian Ocean. The genus *Leiochrides* was formerly defined as having 12 thoracic chaetigers with only capillaries, abdominal chaetigers with only hooks, an indistinct boundary between the thorax and the abdomen, and the absence of branchiae (Augener 1914). However, the generic definition has continuously been modified for several decades. Hartman (1963, 1974) described *L. branchiatus* and *Leiochrides* sp. from California and revealed that some species of *Leiochrides* had transitional chaetigers in the thorax as a unique characteristic that had not been noted in previous studies. The genus *Leiochrides* is represented by having 12 chaetigers with capillary chaetae and only one or two species have the transitional chaetigers with capillary notochaetae and neurohooks. On the other hand, Day (1967) and Fauchald (1977) did not accept the new character proposed by Hartman (1963, 1974) and followed the original generic definition of Augener (1914). Recently, Blake (2000) and Green (2002) reconfirmed the existence of the transitional thoracic chaetigers in *Leiochrides* species and emended the generic definition in the light of Hartman (1963, 1974). We follow the expanded generic definition of Green (2002) in this study.

The genus *Pseudomastus* established by Capaccioni-Azzati and Martin (1992) was defined by the 12 thoracic chaetigers, which comprised 11 thoracic chaetigers with capillaries and the last thoracic chaetiger with capillary notochaetae and neuropodial hooks. The chaetal arrangement of *Pseudomastus* coincides with the expanded generic definition of *Leiochrides* by Blake (2000) and Green (2002). The taxonomic relationship for these two genera, however, has never been precisely defined since the erection of *Pseudomastus* as a new monospecific genus.

This study provides a detailed description of a new species of *Leiochrides* from southern Korea and reveals its morphological distinctiveness through comprehensive comparisons with closely related species. Additionally, the taxonomic status of the genus *Pseudomastus* is discussed.

## Materials and methods

Sediment samples were obtained from the sublittoral muddy-sand bottom of the southern coast of Korea with a 0.05 m^2^ Van Veen grab followed by elutriation on a 0.35 mm sieve in a 30 L seawater container. The remaining organisms on the sieve were transferred to a 1 L collecting jar with a 7% MgCl_2_ solution for anesthesia. The relaxed samples were initially fixed in a 10% formalin solution for an hour before they were preserved in 90% ethanol for subsequent analysis. For the identification of the morphological features, the samples were stained with Shirlastain A (SDLATLAS, Inc.) for three seconds and sorted under a zoom stereomicroscope (Nikon SMZ745T). Line drawings were conducted using a differential interference contrast microscope (Eclipse Ci-L, Nikon) and a digital pen display (Cintiq 22HD, Wacom). The MGSP of the examined specimens were described and photographed as described in Jeong et al. (2017). A Scanning Electron Microscopy (SEM) analysis was carried out to confirm the detailed morphological structure. The specimens were placed in an ultrasonic chamber with distilled water for 30–60 seconds to remove the hoods of the hooded hooks. The treated specimens were dehydrated through a series of increasing concentrations of ethanol ending with two changes of absolute ethanol, followed by critical point drying. The dehydrated specimens were coated with gold and then photographed using the Hitachi S-3000N.

The type materials were deposited in the collections of the Marine Biodiversity Institute of Korea (MABIK) in Seocheon, Korea.

## Systematics

### Family Capitellidae Grube, 1862

#### Genus Leiochrides

Taxon classificationAnimaliaAnnelidaCapitellidae

Augener, 1914

Pseudomastus Capaccioni-Azzati & Martin, 1992: 247–249, figs 1a–h, 2a–f. **Syn. n.**

##### Type species.

*Leiochrides australis* Augener, 1914

##### Type locality.

Western Australia

##### Generic diagnosis

(modified after Green 2002). Thorax with 13 segments including an achaetous peristomium and 12 chaetigers with capillary chaetae. Chaetiger 11 and 12 may have capillary chaetae in both rami or may be transitional with capillary chaetae in notopodia and hooded hooks in neuropodia. Remaining chaetigers with hooded hooks. Parapodia reduced. Retractile branchiae may be present.

#### Leiochrides yokjidoensis sp. n.

Taxon classificationAnimaliaAnnelidaCapitellidae

http://zoobank.org/996ABB3E-3E71-452F-9A3E-BEE6F3015A9D

Figs 2A–F , 3A–H

##### Material examined.

Holotype (complete specimen): MABIKNA00145754, sex uncertain, Yokjido, 34°31.1’N, 128°21.6’E (DDM), subtidal, sandy mud bottom, 53 m depth, April 2016, collector: Man-Ki Jeong. Paratypes (3 incomplete specimens): MABIKNA00145755–NA00145757, same information as holotype.

##### Diagnosis.

Thorax with achaetigerous peristomium and 12 chaetigers. First chaetiger without neuropodia. Chaetigers 1–11 with capillary chaetae only, chaetiger 12 with notopodial capillaries and neuropodial hooks. Abdominal chaetigers with hooded hooks only. Branchiae present on posterior abdominal segments as 2–4 digitate filaments near notopodia. Approximately ten preanal chaetigers without branchiae. Pygidium with four anal cirri.

##### Description.

One complete specimen and eight incomplete specimens. Largest specimen 27 mm long, 0.34 mm wide for 159 chaetigers. Smallest specimens 7 mm long, 0.25 mm wide for 34 chaetigers. Body thread-like, cylindrical, widest in anterior thoracic chaetigers, tapering from abdomen to pygidium. Color in alcohol yellowish or reddish brown.

*Prostomium* short, conical, wider than long, with blunt anterior end; presence of nuchal organs indistinct, eyespots not observed in preserved specimen (Figs 2A–C, 3A). Everted proboscis with numerous short, rounded, papillae, without cilia on tip of papillae (Figs 2C, 3A). Peristomium achaetigerous, wider than long, weakly biannulated and tessellated, subequal or slightly longer than first chaetiger (Figs 2A–C, 3A).

**Figure 1.**
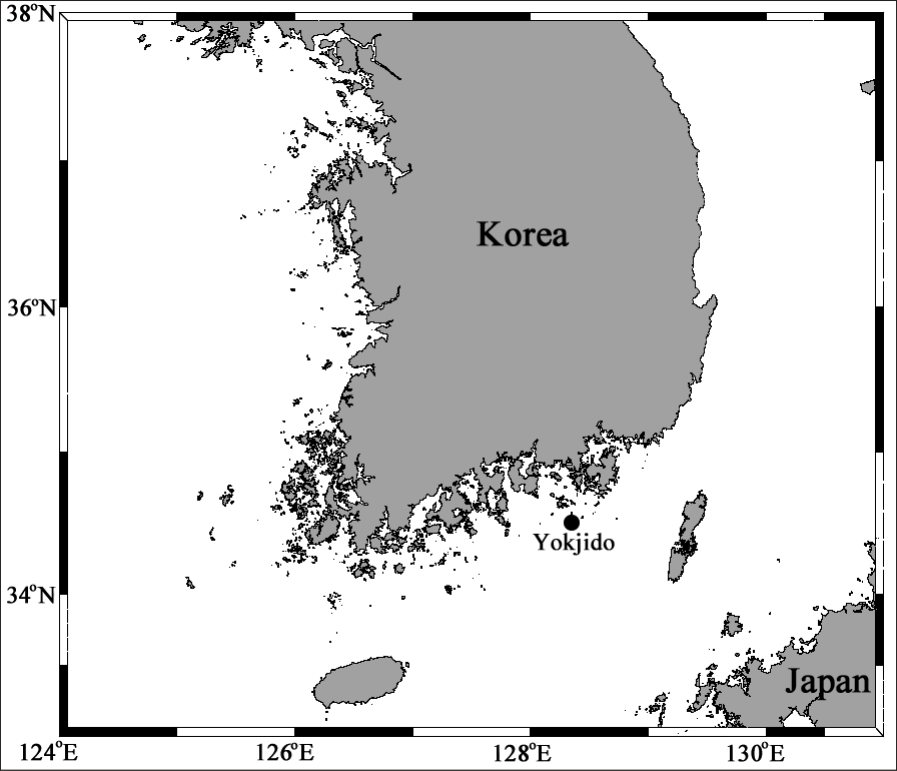
Map of study area with main collection stations indicated.

**Figure 2.**
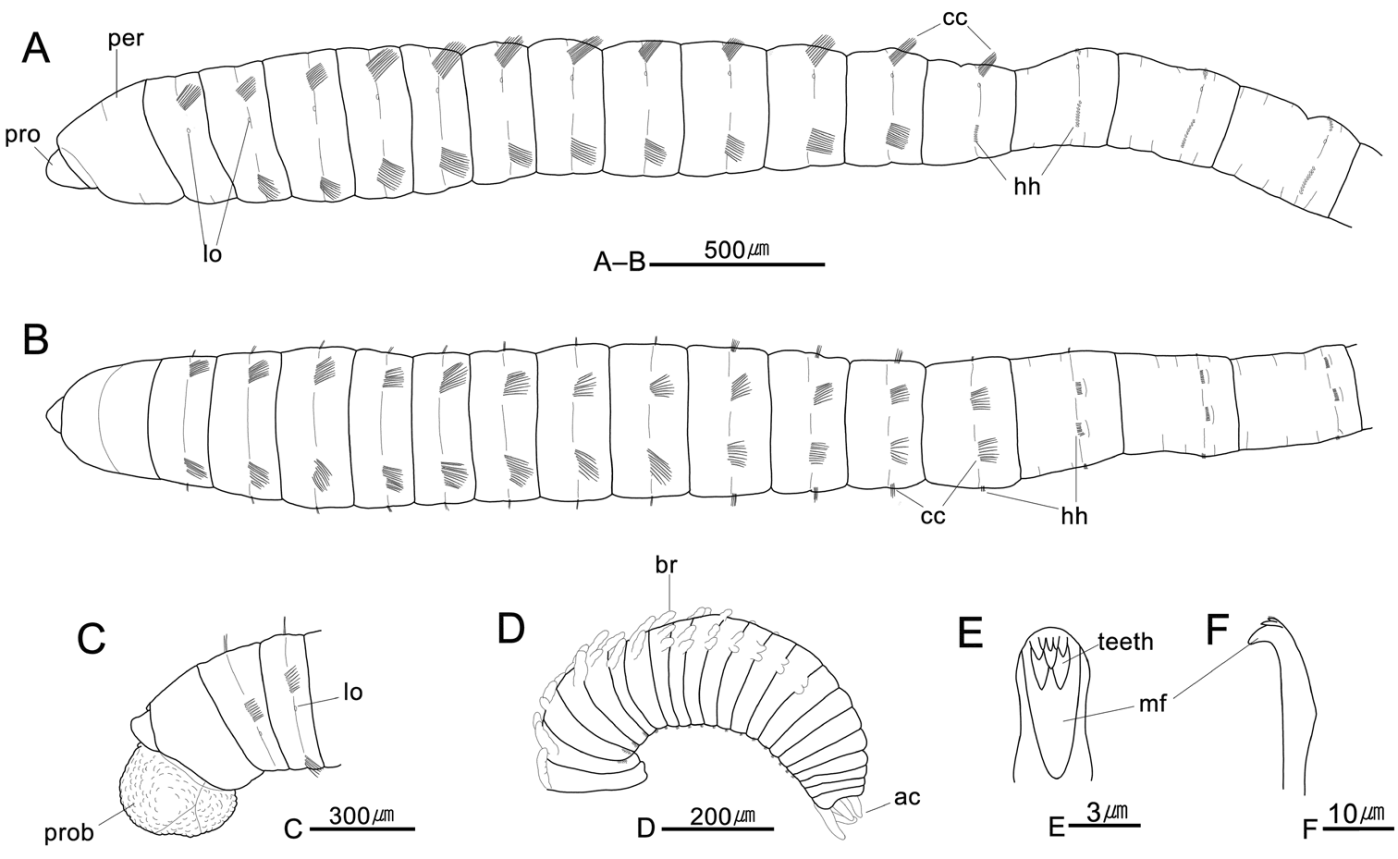
*Leiochrides yokjidoensis* sp. n. **A** anterior end, left lateral view **B** same, dorsal view **C** anterior end with proboscis (MABIKNA00145754), left lateral view **D** posterior end (MABIKNA00145754), left lateral view **E–F** hooded hooks from anterior abdominal notopodium. Abbreviations: ac, anal cirri; br, branchia; cc, capillary chaeta; hh, hooded hook; lo, lateral organ; mf, main fang; per, peristomium; pro, prostomium; prob, proboscis.

**Figure 3.**
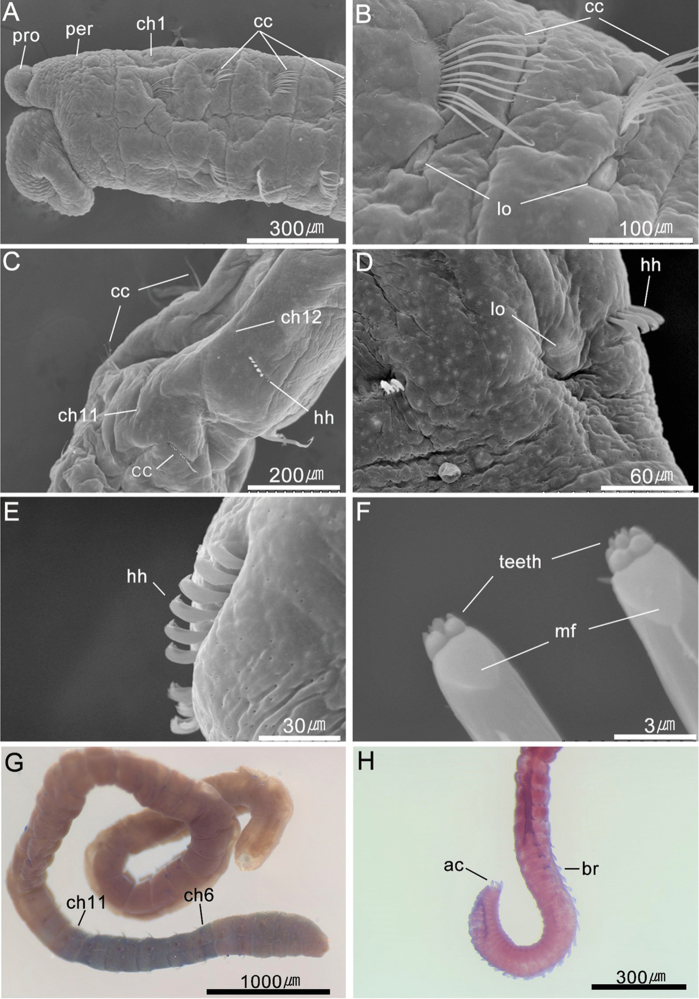
*Leiochrides yokjidoensis* sp. n. **A−F** scanning electron micrographs **A** anterior end in left lateral view **B** chaetigers 4–5 in left dorsolateral view **C** chaetigers 11–12 in left lateroventral view **D** chaetiger 15 in left lateral view **E** notopodial hooded hooks of chaetiger 16 **F** neuropodial hooded hooks of chaetiger 16 in frontal view **G–H** photomicrographs **G** thorax and anterior abdomen, right lateral view showing methyl green staining reaction **H** posterior end of body in left lateral view (MABIKNA00145754). Abbreviations: ac, anal cirri; br, branchia; cc, capillary chaeta; ch, chaetiger; hh, hooded hook; lo, lateral organ; mf, main fang; per, peristomium; pro, prostomium

*Thorax* with 13 segments including single peristomium and 12 chaetigers (Fig. 2A–B). Thoracic segments biannulate, wider than long, with shallow intra- and inter-segmental grooves (Figs 2A–B, 3A). Anterior thoracic segments 1–5 slightly expanded and tessellated. First chaetiger uniramous with only notochaetae, both parapodia of chaetigers 1–11 and notopodia of chaetiger 12 each with 8–12 capillaries per fascicle; neuropodia of chaetiger 12 with 8 hooded hooks per fascicle (Fig. 2A). All capillary chaetae unilimbate, with narrow wing, whip-like, broad basally, and narrow apically; dorsal notochaetae without spinules on distal region of chaeta (Fig. 3B). Notopodia dorso-lateral and neuropodia ventro-lateral (Fig. 2A–B). Lateral organs distinct, oval shape, not protruding; present between notopodia and neuropodia, nearer to notopodia (Figs 1A, 2B, D). Genital pores not observed.

*Transition* between thoracic and abdominal region distinguished by change in position and type of chaetae and length of segments; abdominal segments longer and slightly narrower than thoracic segments, gradually smaller posteriorly; notopodial hooded hooks first present on chaetiger 13 (first abdominal chaetiger) (Fig. 2A–B). Abdominal chaetigers with hooded hooks on posterior end of segment; thoracic chaetigers with capillary chaetae or neuropodial hooks in center of segment (Fig. 2A–B).

*Abdominal parapodial lobes* slightly developed, located in posterior half of segment, well separated from each other; parapodial lobe gradually reduced posteriorly (Fig. 2A–B, D). Abdominal notopodia with 5–6 hooded hooks per fascicle; neuropodia with 8–10 hooded hooks per fascicle, 2–3 hooks on terminal segments (Fig. 2A–B).

*Hooded hooks* short, with main fang extending slightly beyond hoods; hood flared; shaft slightly enlarged like manubrium (Figs 2F, 3E). Hooks with three rows of small teeth above main fang; two in basal row, three in middle row, and 3–5 in superior rows (Figs 2E, 3F).

*Branchiae* digitiform, cylindrical, retractile; abdominal chaetiger 120–150 each with 3–5 branchiae per fascicle emerging from notopodia which lack hooks in this region; eight preanal 8 segments without branchiae (Figs 2D, 3H). Pygidium an oval ring, with four digitate caudal cirri (Figs 2D, 3H).

##### Methyl green staining pattern.

Prostomium not stained. Peristomium and thoracic chaetigers 1–5 slightly stained in blue but rapidly fades. Chaetigers 6–9 stained blue, with narrow transverse blue speckled band near intra-segmental furrow (Fig. 3G). Post-chaetal region of chaetiger 10 and pre-chaetal region of chaetiger 11 with intense blue speckles on epidermis (Fig. 3G). Abdominal segments without distinct staining pattern.

##### Etymology.

The new species is named for its occurrence in Yokjido, Korea.

##### Distribution.

*Leiochrides yokjidoensis* sp. n. is distributed in the subtidal habitat (53 m) of the southern part of Korea.

**Ecology.** The surface sediment is mainly composed of sandy mud with fragmented shells. *Mediomastus* Hartman, 1944 and *Notomastus* M. Sars, 1851, also belonging to the Capitellidae, also occurred at the same location.

##### Remarks.

*Leiochrides yokjidoensis* sp. n. is distinct in the morphological combination of 12 thoracic chaetigers, chaetigers 1–11 with only capillary chaetae, and the last thoracic chaetiger with the notopodial capillaries and neuropodial hooks. Among the genera of Capitellidae the presence of 12 thoracic chaetigers and neurohooks in last thoracic chaetiger are shared with *Leiochrides*, *Pseudomastus*, and *Scyphoproctus* Gravier, 1904. However, *Scyphoproctus* is clearly separated from the new species by the presence of the unique anal plaque, acicular spines in the posterior abdomen, and two achaetous segments in the anterior part of the thorax. In this study, the new species was placed under the genus *Leiochrides*, because the generic diagnosis of *Pseudomastus* mostly agreed with that of *Leiochrides* (see details in discussion section). *Leiochrides yokjidoensis* sp. n. closely resembles *L. hemipodus* and *P. deltaicus* in the presence of the neurohooks in the thorax, the uniramous first chaetiger, the two distinct basal teeth above the main fang, and the presence of the branchiae in the posterior abdomen (Table 1). In particular, *L. yokjidoensis* sp. n. and *P. deltaicus* correspond in the presence of multiple cirri on the pygidium (Table 1). However, the new species is discriminated from *P. deltaicus* by the following morphological characteristics: the absence of eyespots in the preserved materials, dorsal notochaetae without distal spinules, a proboscis without cilia on the tip of the papillae, the number of capillary chaetae (8–12 vs. 13–20) and neurohooks (8–10 vs. 11–12) per fascicle, the number of teeth above the main fang (8–10 vs. 7), the absence of neuropodial hooks in chaetiger 11, and the number of anal cirri (4 vs. 3). On the other hand, the holotype of *L. hemipodus* has identical chaetal arrangement to *L. yokjidoensis* sp. n., but it differs in the number of teeth above the main fang (3 vs. 8–10), the maximum number of branchiae per fascicle (12 vs. 5), and the species-specific MGSP (chaetigers 1–12 vs. 6–11, Table 1). In addition, *L. hemipodus* has been reported only in deep basin near California, but *L. yokjidoensis* sp. n. was found in shallow waters of southern Korea (Table 1). The paratype of *L. hemipodus* has a different chaetal arrangement (chaetigers 11–12 with neuropodial hooks) to the holotype of *L. hemipodus*, however these type specimens were almost identical in the remaining characteristics (Blake 2000, Green 2002, Table 1). Generally, capitellids have developmental variation in the chaetal arrangement on the thorax, and the hooded hooks are replaced by capillaries with development (Fredette 1982, Blake 2009). Thus, the differences in chaetal arrangement between the type specimens of *L. hemipodus* may be associated with the developmental variation within a species. Blake (2000) had reported *L. hemipodus* from Santa Maria basin (603 m) and compared his specimens with the characteristics of the paratype of *L. hemipodus* Hartman, 1960. According to his description, *L. hemipodus* also differs from *L. yokjidoensis* sp. n. in the presence of a band of glands on chaetiger 6 and the shape of the branchiae (Table 1).

**Table 1.** Morphological comparison between *L. yokjidoensis* sp. n. and most related species. A: absent; P: present; C: capillary chaeta; H: hooded hook; L: length; W: width; Ch: chaetiger; incomp: incomplete specimen; no: notopodia; neu: neuropodia; NM: not mentioned.

Species	Overall size (L/W, mm)	Eyes	Thoracic chaetal arrangement	No. of chaetae per fascicle		Dental structure of hooks	Special character	No. of branchiae per fascicle	No. of anal cirri	Methyl green staining pattern	Locality	References
				Thorax	Abdomen							
*L. hemipodus* (Holotype)	30–40/1–2 (incomp.)	A	no: 12C neu: 11C+1H	NM	NM	2 rows (2/1)		3–12 (sometimes bifurcated)	NM	Ch 1–12	San Pedro basin, California (887m)	Hartman (1960); Green (2002)
*L. hemipodus* (Paratype & its related sp.)	?/1	A	no: 12C neu: 10C+2H	NM	NM	2 rows (2/1)	Glands on chaetiger 6	ca. ~15 (in fig. 4.9; branched)	0	Ch 1–12	Santa Maria basin, California (603m)	Blake (2000)
*L. yokjidoensis* sp. n.	27/0.34	A	no: 12C neu: 11C+1H	8–12(C) 8(H)	no: 5–6(H) neu: 8–10(H)	3 rows (2/3/3–5)		3–5 (palmate)	4	Ch 6–11	Korea (~43m)	This study
*P. deltaicus*	50/0.7	P	no: 12C neu: 10C+2H	13–20(C) 12(H)	11–12(H)	3 rows (2/1/4)	Distal spinules on notopodial capillaries	2–4	3	NM	Spain (~6m)	Capaccioni-Azzati and Martin (1992)

## Discussion

Capaccioni-Azzati and Martin (1992) established the genus *Pseudomastus* to accommodate the only species, *P. deltaicus* characterized by 12 thoracic chaetigers, the first chaetiger with only notochaetae, chaetigers 1–10 with capillaries, the last two thoracic chaetigers with notopodial capillaries and neuropodial hooks, the posterior end of each notopodium with 2–4 digitate branchiae on chaetigers 205–210 to chaetigers 255–260, and the pygidium with three anal cirri. At this point, the genus *Leiochrides* was earlier assigned with morphological characteristics that coincided with *Pseudomastus*. However, Capaccioni-Azzati and Martin (1992) did not detect the close relationship in morphology between *Leiochrides* and their new genus, *Pseudomastus*. Hartman (1963, 1974) had proposed the presence of transitional chaetigers, with capillary notochaetae and neurohooks, as an important characteristic of *Leiochrides* but it was not accepted as a generic diagnosis by Fauchald (1977). Blake (2000) later added this characteristic to the generic definition, which was redefined as 12 chaetigers with capillary chaetae but sometimes one or two of the chaetigers having notopodial capillaries and neurohooks. According to this expanded generic definition, the characteristics of *Pseudomastus* also matched those of *Leiochrides*. In addition, the uniramous first chaetiger, the multiple branchiae in the posterior part of abdomen, the two distinct basal teeth above the main fang of the hooks, and the presence of multiple anal cirri are also identical features that are found in some members of *Leiochrides*. Although *P. deltaicus* is unique in having spinules on the distal edge of the dorsal capillary notochaetae and cilia on the papillae of the proboscis, these seem to be species level characteristics within the Capitellidae. Thus, we regard the monospecific *Pseudomastus* as a junior synonym of *Leiochrides*.

This study provides the detailed morphological features of *L. yokjidoensis* sp. n. including the presence/absence of the lateral organ and genital pore, the number of chaetae per fascicle, MGSP, and the number of anal cirri. Green (2002) used variations in the thoracic chaetal distribution and the details of the hooded hooks among six of the described *Leiochrides* species in an identification key to species. Nevertheless, the use of additional taxonomic information on the distribution of the lateral organs, nephridia or genital pores, MGSP, and characteristics of the pygidium are still insufficient to distinguish many of the previously described *Leiochrides* species, including *L. hemipodus*. Therefore, a precise examination of the morphological details and a re-examination of existing *Leiochrides* species is needed in the future and may prove useful in a clarification of the taxonomic relationship between *Leiochrides* species.

### Key to species of genus *Leiochrides* (modified from Green 2002)

**Table:** 

1	First chaetiger biramous; chaetigers 1–12 with capillary chaetae only	**2**
–	First chaetiger uniramous	**4**
2	Prostomium deeply bifid; hooded hooks with small teeth above main fang in 3 rows	***L. biceps***
–	Prostomium without dorsal furrow; hooded hooks with small teeth above main fang in 2 rows	**3**
3	Hooded hooks with 4–6 teeth above main fang	***L. africanus***
–	Hooded hooks with 8–10 teeth above main fang	***L. pallidior***
4	Chaetigers 1–12 with capillary chaetae	**5**
–	One or more transitional chaetigers with capillary notochaetae and neurohooks	**7**
5	First two abdominal segments with notopodial capillaries; pygidium with 4 anal cirri; branchiae present	***L. norvegicus***
–	Abdominal segments without capillary chaetae	**6**
6	Hooded hooks with 5 teeth above main fang in 3 rows	***L. andamanus***
–	Hooded hooks with 10–11 teeth above main fang in 3 rows	***L. australis***
7	Chaetiger 12 transitional with capillary notochaetae and neurohooks	**8**
–	Chaetigers 11–12 transitional with capillary notochaetae and neurohooks	**9**
8	Hooded hooks with 8–10 teeth above main fang in 3 rows; posterior abdomen with 3–5 branchiae per fascicle; pygidium with 4 anal cirri	***L. yokjidoensis* sp. n.**
–	Hooded hooks with 3 teeth above main fang in 2 rows; posterior abdomen with 3–12 branchiae per fascicle; pygidium without anal cirri	***L. hemipodus***
9	Hooded hooks with 3 teeth above main fang in 2 rows	***L. branchiatus***
–	Hooded hooks with 7 teeth above main fang in 3 rows; posterior abdomen with 2–4 branchiae per fascicle; pygidium with 3 anal cirri	***P. deltaicus***

## Supplementary Material

XML Treatment for Leiochrides

XML Treatment for Leiochrides yokjidoensis
